# Trans-Fistula Anorectoplasty (TFARP): Our Experience in the Management of Anorectovestibular Fistula in Neonates

**Published:** 2012-07-01

**Authors:** Ashrarur Rahman Mitul, K M N Ferdous, Md. Shahjahan, Jaglul Gaffar Khan

**Affiliations:** Department of Pediatric Surgery, Dhaka Shishu (Children) Hospital, Dhaka.; 1 Dhaka Medical college and Hospital Dhaka, Bangladesh.

**Keywords:** Neonates, anorectal malformation, vestibular fistula, trans-fistula anorectoplasty

## Abstract

Aim: The purpose of the study was to observe the outcome of trans-fistula anorectoplasty (TFARP) in treating female neonates with anorectovestibular fistula (ARVF).

Methods: A prospective study was carried out on female neonates with vestibular fistula, admitted into the surgical department of a tertiary level children hospital during the period from January 2009 to June 2011. TFARP without a covering colostomy was performed for definitive correction in the neonatal period in all. Data regarding demographics, clinical presentation, associated anomalies, preoperative findings, preoperative preparations, operative technique, difficulties faced during surgery, duration of surgery, postoperative course including complications, hospital stay, bowel habits and continence was prospectively compiled and analyzed. Anorectal function was measured by the modified Wingspread scoring as, “excellent”, “good”, “fair” and “poor”.

Results: Thirty-nine neonates with vestibular fistula underwent single stage TFARP. Mean operation time was 81 minutes and mean hospital stay was 6 days. Three (7.7%) patients suffered vaginal tear during separation from the rectal wall. Two patients (5.1%) developed wound infection at neoanal site that resulted in anal stenosis. Eight (20.51%) children in the series are more than 3 years of age and are continent; all have attained “excellent” fecal continence score. None had constipation or soiling. Other 31 (79.5%) children less than 3 years of age have satisfactory anocutaneous reflex and anal grip on per rectal digital examination, though occasional soiling was observed in 4 patients.

Conclusion: Primary repair of ARVF in female neonates by TFARP without dividing the perineum is a feasible procedure with good cosmetic appearance and good anal continence. Separation of the rectum from the posterior wall of vagina is the most delicate step of the operation, takes place under direct vision. It is very important to keep the perineal body intact. With meticulous preoperative bowel preparation and post operative wound care and bowel management, single stage reconstruction is possible in neonates with satisfactory results.

## INTRODUCTION

Anorectal malformation (ARM) is a well recognized condition since antiquity and represent a wide spectrum of defects. Worldwide incidence is 1 in 5000 live births [1]. ARVF is the commonest ARM in female children [2]. Pena and deVries in 1982 reported posterior sagittal anorectoplasty (PSARP) as an operative procedure for high or intermediate imperforate anus. Okada et al devised anterior sagittal anorectoplasty (ASARP) for repair of ARVF [3, 4]. Procedures without colostomy have been described by different authors like anal transposition [5] repair of ARVF without opening the fourchette [6], repair of vestibular and perineal fistula, [7] Technical variations in single-stage methods have been described in different series with satisfactory results [8-16].

Post-operative complications such as wound infection, wound dehiscence with fibrosis, subcutaneous leak, skin suture dehiscence, pelvic floor descent, anal stenosis, rectal prolapse, recurrence of fistula, soiling, incontinence, constipation and unsatisfactory cosmetic outcome have been described in different single stage procedures [8-14]. These complications can be reduced considerably repairing by trans-fistula anorectoplasty (TFARP) [16].

## MATERIALS AND METHODS

This was a prospective study done from January 2009 to June 2011. Ethical permission was taken from the ethical committee. We included all female neonates diagnosed ARVF for single-stage TFARP. Detailed history and clinical examination was carried out including the perineum, buttocks, spine and other systems for associated anomalies. An informed written consent for primary one stage procedure was taken and option was given to choose between single stage and multistage procedure.

**Preoperative preparation:** All patients underwent dilatation of the fistula with simple rubber catheter of size 8-10 Fr and rectal washouts with normal saline four times in a day, beginning 48 hours pre-operatively or earlier in the presence of constipation and abdominal distension. Routine blood investigations such as hemogram, renal function tests, serum electrolytes followed by ultrasonography of abdomen and pelvis to rule out genitourinary anomalies was done. X-ray whole body and spine was done to exclude other bony anomalies and a 2-D echo was done pre-operatively in all patients.

**Surgical technique: **The choice of anesthesia was general with caudal block. Intravenous cephradine, gentamycin and metronidazole were given at induction. Surgery was performed in lithotomy position after catheterizing the bladder. Peri-fistula traction sutures were taken followed by peri-fistula incision with electrocautery which were deepened and circumferential dissection was carried out in a plane so as not to damage the rectal wall or the vaginal wall anteriorly. Anterior dissection extended upto cervix and posteriorly upto the sacral promontory. No incision was made over the perineum and perineum was kept intact. Proposed anal site was determined by the anal dimple and confirmed by the use of a muscle stimulator. A vertical incision of about 2cms was made at that site (Figure 1), and opening created in the external sphincter complex, through which mobilized rectum was pulled and fixed to the deep muscle complex, with vicryl 4’0. Anoplasty was done with 12 stitches with 4’0 vicryl; the neoanus allowed 10/12 sized Hegar’s dilator. The vestibular wound was closed with 4’0 vicryl interrupted stitches. The immediate post operative picture is shown in Figure 2. The rectum was packed with vaseline gauze after surgery which was removed on the next day.

**Figure:**
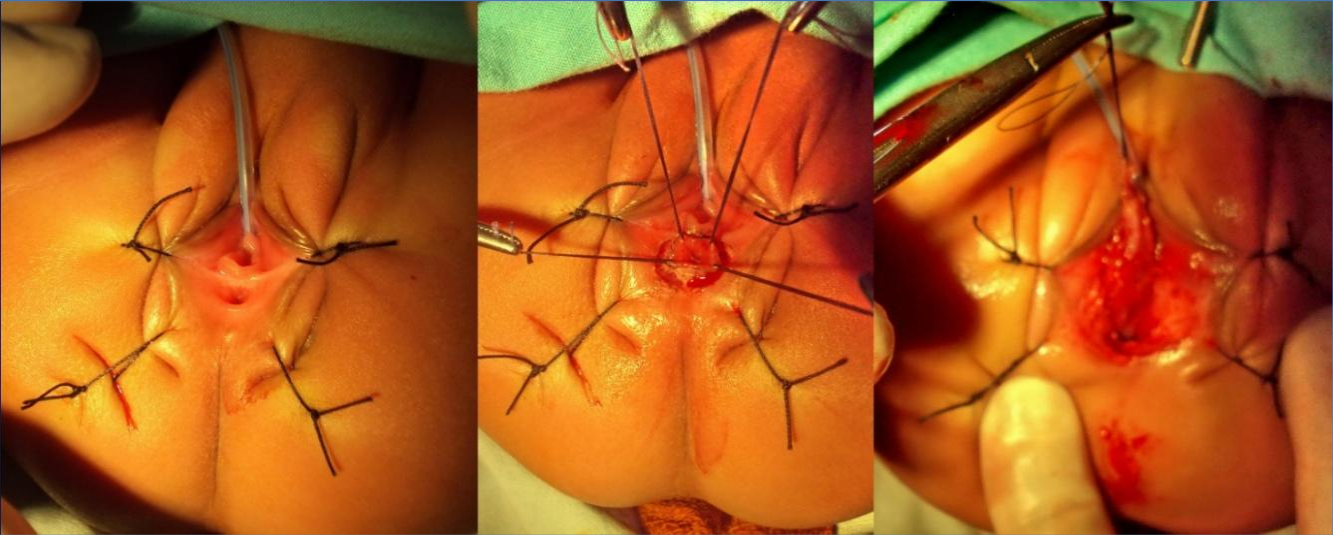
Figure 1: Vestibular fistula- various step of mobilization of fistula along with rectum. Incision was also given at the proposed anus site.

**Figure:**
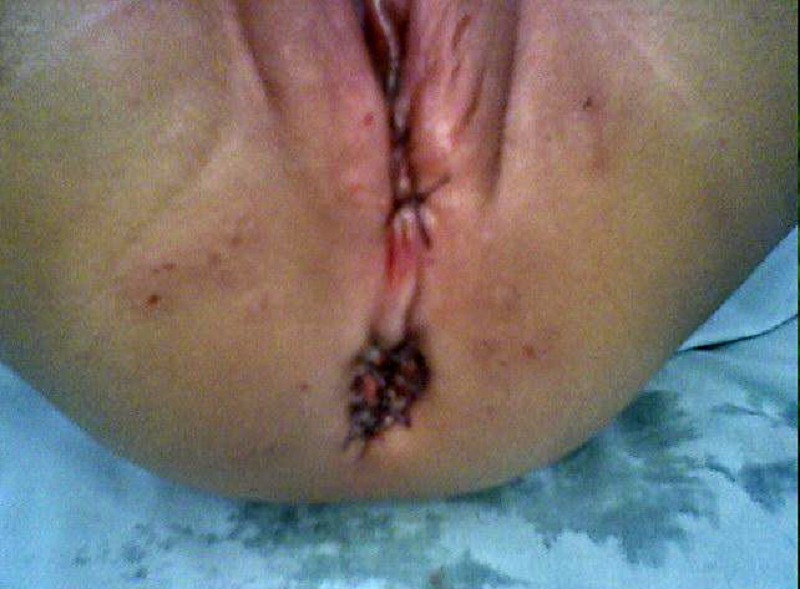
Figure 2: Immediately postoperative figure.

**Postoperative care:** Urethral catheter was kept in situ for up to the 5th postoperative day. The mother was instructed to apply povidone iodine solution over the operated wound and neoanus several times a day, and after each bowel movement, antibiotic ointment was applied three to four times a day. Patients were allowed breast feeding on the 1st post operative day. Intravenous antibiotics were continued for up to the 5th postoperative day. Majority of the patients were discharged on the 6th postoperative day unless complications occurred, when the stay was prolonged. Anal dilatations were started on 14th post-operative day with Hegar’s dilator, taught to the parents who were asked to regularly dilate for two times a day for two weeks, once daily for one month, twice a week for one month, once a week for one month, and then once a week for three months.

**Follow up:** All patients are under regular follow up; the period of assessment ranges from 11months to 3 and half yrs till date. Follow up schedules were 14th postoperative day, monthly for one month, three monthly for one year, and yearly thereafter. During each visit following points were noted: appearance, site and size of the neoanus, condition of the wound. Data regarding early (upto 3 months) complications like wound infection, wound dehiscence, skin excoriation, and delayed (3 months to 3 years) complications like, mucosal prolapse, fistula formation, stenosis was collected. Information about whether scheduled dilatation was followed, bowel habits, continence, soiling was gathered. Anocutaneous reflex and anal squeeze on per rectal digital examination were performed for younger children who had not attained the age for continence (less than 3 years) and fecal continence score for those three years or more were applied according to modified Wingspread score where operative outcome is designated as “excellent”, “ good”, “fair “ and “ poor” [18].

## RESULTS

The mean age of 39 operated patients was 14.9 days (range: 5-28 days). Six (15.4%) patients had constipation and 2 (5.1%) had abdominal distension. The associated anomalies of the patients are shown in Table 1.

**Figure:**
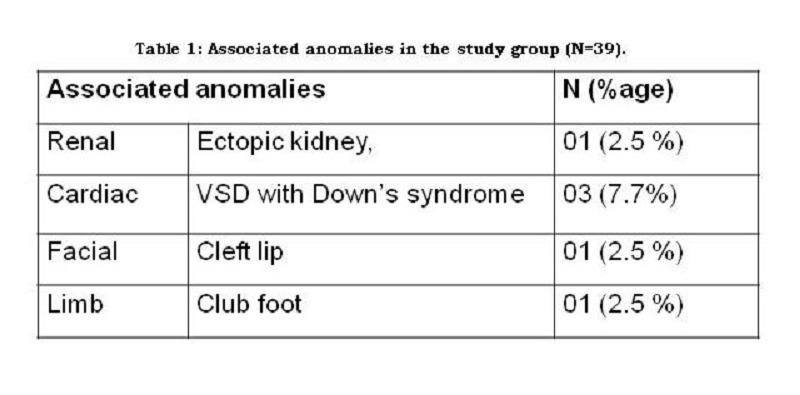
Table 1

The mean operation time was 81.2 minutes (SD ± 4.8). 03 (7.7%) patients suffered vaginal wall tear during separation of rectal wall from vaginal wall. Two (5.1%) girls developed infection at neoanal site leading to anal stenosis later. The infections improved with conservative treatment. Anal stenosis was treated with anal dilatations with Hegar’s dilator.

Thirty seven (94.9%) patients were discharged on 6thday postoperative day; 2 patients with wound infection stayed for 8-10 days. Of the 39 patients, 8 patients (20.51%) who are now 3 years or older are toilet trained and have satisfactory voluntary bowel movements and, not constipated, without soiling. The younger children have bowel pattern as per age, though do not pass in the toilet, but the stool pattern does not reveal any problem like constipation. Only 4 of them have occasional soiling. They have satisfactory anal contraction on stroking the perianal skin and good anal grip on per rectal digital examination, with an average of 2-5 bowel movements per day depending on age. The cosmetic outcome was satisfactory (Fig.3). Table 2 shows the bowel habits of all the patients.

**Figure:**
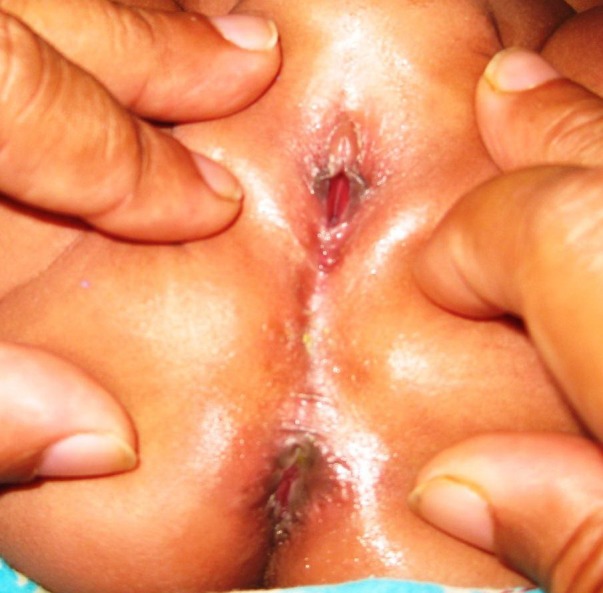
Figure 3: Cosmetic result at three months of operation.

**Figure:**
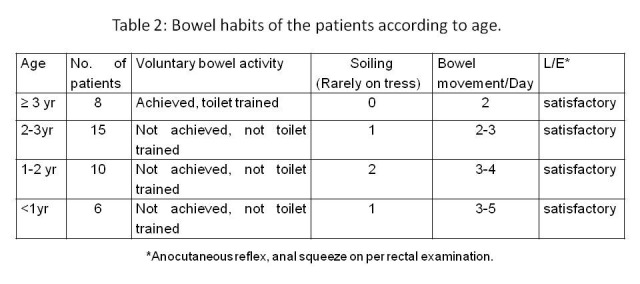
Table 2

## DISCUSSION

Ideally, all ARM should be repaired in the neonatal period. There is evidence that the somato-sensory inputs from the perianal skin which is an important component of continence, is lost if unused for more than three to four months. By early usage the perineal musculature is trained early, and synapses and neuronal networks may be formed which may enhance the chance of normal or near normal functions. When patients present late, this advantage is clearly unattainable [9,15,19]. Early restoration of gastrointestinal continuity is very important to establish the brain-defecation reflexes early [12]. Traditionally, ARMs were reconstructed with a protective colostomy because of the fear of wound healing and subsequent loss of anal sphincter complex with the risk of impairment of future continence. The belief that a protective colostomy may prevent wound infection is questionable [9]. Although Pena prefers the use of a colostomy followed by limited PSARP in case of ARVF [17], the primary advantages of performing anorectoplasty without a colostomy are the avoidance of colostomy-related complications and multi-staged surgeries [19,20].

Currently, the popularity of single staged procedures for almost all types of ARMs is increasing [7,9,21]. These advances have been possible due to development of science and technology and increased surgical expertise.

Single-stage procedure not only attains the same results as the staged procedure with fewer early operative complications, but it also obviates the risks of multiple anaesthesias and multiple operations. It eases the physiologic, psychologic and economic burden [10]. Colostomy formation always done in emergency settings is associated with its own set of complications and cannot be considered as a minor surgery. Colostomy related complications range from 28% to 74% [10]. Of these, peristomal excoriation, prolapse, parastomal hernias, leakages, intra abdominal adhesions or even worse, bowel obstruction are most common [9]. Similarly, colostomy closure is still not a very simple surgery, and carries significant morbidity and mortality [20].

ARVF is the commonest form of ARM in girls and is associated with the best prognosis. It is estimated that 93% of patients with ARVF will develop voluntary bowel movements [7]. Incidences of incontinence and soiling following single stage anorectoplasty range from 1.9% - 47.9% among different procedures in different series [5-7,10], whereas few had no incidence of incontinence or soiling [9,13,14]. Surgical correction of ARVF has been performed by various techniques for many years [2-16]. The anterior sagittal anorectoplasty (ASARP) and limited posterior sagittal anorectoplasty (PSARP) involve partial division of the anterior margin of the external sphincter and levator muscle respectively [3, 4]. The technique of TFARP described above differs from the standard limited PSARP and the other procedures in that

1. The external sphincter complex and the levator muscle are not divided;
2. Perineal or posterior sagittal incisions are not utilized, and therefore the perineal body and neurovascular supply are not disturbed;
3. The rectal pouch is not tapered, and hence the internal sphincter is preserved [16].

We performed the surgery with the patient in lithotomy position for better delineation of the anatomy, resulting in more accurate operation followed by improved surgical outcome. The absence of any incision over the perineum leads to good cosmetic and functional result and reduces the risk of wound dehiscence in this critical region [16]. Correct placement of the rectum through the external sphincter is vital in TFARP for continence. And if the neoanus is not placed through the center of the external sphincter, the child may be incontinent. Taking incision on skin where external sphincter fibers are seen with use of muscle stimulator shows contraction of the sphincter in this procedure. Thus it is possible to place the rectum directly in the center of the external sphincter muscle complex. TFARP thus provides satisfactory functional and cosmetic results with a normally situated anal opening.

In different reports of single-stage surgery, wound infection rate ranged from 0% [6, 16] to 5.7%-10.6% [5,7,9,13]. In our series, the wound infection rate and resultant anal stenosis was 5.1% that corroborates with the reported incidence of 2.9% - 6.8% [5-10,16,17]. Several authors report excellent continence ranging from 90.5 -100% were continent [5,7,13,14]. In other series, continent scores were excellent or good among 58.3%, - 72% and fair in 20% [6,10]. In our study 8 patients have attained the age of continence and all are having excellent score. The rest are having bowel habits as per their age. Though constipation has been found to be a significant long-term problem ranging from 3.7% to 47.9% children, some of them required dilatation, laxatives and enema on occasion [7-12,16,18]; we have not encountered this condition in our series.

## Conclusion

Single stage TFARP for the repair of the vestibular fistula is a feasible procedure even in neonates. Continence, soiling, cosmetic outcome, and anal stenosis are comparable to other procedures. The correct placement of the rectum through the external sphincter, keeping the components of fecal continence intact, minimal tissue handling, keeping the perineum intact, are the important steps of the surgery. Adequate pre and post operative bowel management, proper wound care and dilatation programs are vital for satisfactory outcome.

## Footnotes

**Source of Support:** Nil

**Conflict of Interest:** None

